# Enhancing Biocide Safety of Milk Using Biosensors Based on Cholinesterase Inhibition

**DOI:** 10.3390/bios15010026

**Published:** 2025-01-06

**Authors:** Lynn Mouawad, Georges Istamboulie, Gaëlle Catanante, Thierry Noguer

**Affiliations:** 1 Biosensors Analysis Environment Group (BAE-LBBM), Université de Perpignan, Via Domitia, 52 Avenue Paul Alduy, Cedex, F-66860 Perpignan, France; lynn.mouawad@univ-perp.fr (L.M.); georges.istamboulie@univ-perp.fr (G.I.); 2 Laboratoire de Biodiversité et Biotechnologie Microbienne (LBBM), Sorbonne Université, Observatoire Océanologique, F-66650 Banyuls-sur-Mer, France

**Keywords:** quaternary ammoniums, biocides, cholinesterases, biosensor

## Abstract

A sensitive and reliable electrochemical biosensor for the detection of benzalkonium chloride (BAC) and didecyldimethylammonium chloride (DDAC), the most commonly used disinfectant biocides in the agri-food industry, is described. Acetylcholinesterase from *Drosophila melanogaster* (DM AChE) and butyrylcholinesterase from horse serum (BChE) were immobilized by entrapment in a photocrosslinkable polymer on the surface of carbon screen-printed electrodes. Preliminary tests conducted in phosphate buffer showed limits of detection (LODs) of 0.26 µM for BAC using the BChE-based sensor and 0.04 µM for DDAC using the DM AChE sensor. These performances comply with the European regulation for dairy products, which sets a maximum allowable concentration of 0.28 µM for biocides. However, when tested directly in milk samples, a dramatic decrease in the sensitivity of both sensors towards BAC and DDAC biocides was reported. To overcome this problem, a simple liquid–liquid extraction was necessary prior to biosensor measurements, ensuring that the biosensors met European regulatory standards and provided an unbiased response.

## 1. Introduction

Quaternary ammonium salts (QAs) are surfactants containing a quaternary cationic nitrogen atom. Their structures and properties vary based on the nature of the radicals bonded to the nitrogen atom [1]. Benzalkonium chloride (BAC) and didecyldimethylammonium chloride (DDAC) are generally used as antimicrobial disinfectants [2].

In the agri-food industries, the disinfection of microorganisms is necessary; therefore, BAC and DDAC are commonly used for this purpose. However, in the case of poor rinsing after disinfection, residues of these compounds persist on surfaces and become potential contaminants in food. The presence of these compounds in food is harmful to consumers, causing various health problems ranging from gastrointestinal problems to cancer [3]. For this reason, the European Union (EU) has regulated that the maximum residue limit (MRL) of both BAC and DDAC in dairy products and many food samples is 0.1 mg/kg or 0.28 µM.

In 2012, various food corporations informed the European Commission (EC) of the presence of non-negligible amounts of BAC and DDAC in food [4]. In fact, BAC was found at rates of 19 mg/kg in ice cream samples, while the maximum residue limit allowed by the EU is 0.1 mg/kg [5].

In the case of milk, quaternary ammonium compounds are used, particularly for teat disinfection, and because of their tendency to adhere to the surface of the teat, they are often transferred to the milk. Various conventional analytical techniques have been described for the detection of QAs in dairy products. LC-MS/MS has been used to detect BAC and DDAC biocides in raw milk, milk powder, hard-pressed cheese, and processed cheese. The limits of quantification (LOQ) recorded by this method ranged from 5 μg·kg^−1^ to 35 μg·kg^−1^ depending on the analytes [6]. Despite achieving an excellent performance, the described technique has several limitations, including a high cost, time consumption, and the necessity of trained personnel [7]. Because of the increased use of QAs (most commonly BAC and DDAC) and considering the high probability of finding them in food samples following their transfer from disinfected surfaces, it is important to pave the way for alternative methods for the sensitive and cost-effective detection of these compounds, particularly in milk. Based on a report showing the inhibitory effect of quaternary ammoniums on electric eel acetylcholinesterase, we recently described a biosensor that allows for the detection of QAs in water with acceptable sensitivity and selectivity [8].

The objective of this study was thus to develop a cholinesterase-based biosensor capable of detecting BAC and DDAC biocides in milk. For this purpose, two biosensors were designed based on different cholinesterase enzymes: acetylcholinesterase from *Drosophila melanogaster* (DM AChE) and butyrylcholinesterase from horse serum (BChE). Because of the complexity of milk matrices, three types of cow milk generally marketed for human consumption were investigated: whole milk (WM) (>3.5% fat), partially skimmed milk (PSM) (1.5–1.8% fat), and skimmed milk (SM) (<0.5% fat) [9]. To the best of our knowledge, this is the first time that a biosensor has been used for the detection of QAs in milk.

## 2. Materials and Methods

### 2.1. Materials

The production of *Drosophila melanogaster* acetylcholinesterase (DM AChE) was carried out by the Centre de Recherche de Biochimie Macromoléculaire (CRBM) in Montpellier, France. Butyrylcholinesterase (BChE) from horse serum, acetylthiocholine chloride and iodide, Ellman’s reagent, 5,5′-dithiobis(2-nitrobenzoic acid) (DTNB), and the quaternary ammonium biocides benzalkonium chloride (BAC, C12-C18) and didecyldimethylammonium chloride (DDAC) were purchased from Sigma-Aldrich (St. Louis, MO, USA). For cholinesterase enzyme immobilization, Biosurfine-MRH photopolymer (PVA) was purchased from Toyo Gosei Kogyo Co. (Chiba, Japan). Polyvinyl chloride (PVC) sheets measuring 200 mm × 100 mm × 0.5 mm were acquired from SKK (Denzlingen, Germany) to serve as supports for the screen-printed electrodes. The Graphite ink (Electrodag 423SS) and silver/silver chloride ink (Electrodag 6037SS) used for screen printing were provided by Acheson (Plymouth, UK). Cobalt phthalocyanine (CoPC)-modified carbon paste was sourced from Gwent Electronic Materials, Ltd. (Gwent, UK), while glycerophtalic paint, used as an insulating layer, was obtained from AkzoNobel (Montataire, France). Ethyl acetate 99.7% HPLC grade was purchased from Sigma-Aldrich (St. Louis, MO, USA), and acetonitrile HPLC 99.9% grade was purchased from VWR France.

### 2.2. Methods

#### 2.2.1. Determination of Cholinesterase Enzyme Activity

Cholinesterase activity was measured by Ellman’s method in PBS at 0.1 M and pH 7, using a Shimadzu UV-1800 spectrophotometer (Marne-la-Vallée, France). Enzymatic kinetics were carried out in the presence of acetylthiocholine iodide (AtCh) at 0.01 M. Upon the addition of cholinesterase, ATCh is hydrolyzed into thiocholine, which undergoes dimerization in presence of 0.1 M 5,5′-dithiobis-2-nitrobenzoic acid (DTNB), leading to the production of 5-thio-2-nitrobenzoic acid (TNB) [10]. The absorbance of this yellow product (ε = 14,150 M^−1^ cm^−1^) is measured continuously at 412 nm over 1 min, allowing for the determination of the reaction velocity. One enzyme unit (U) was defined as the amount of enzyme that allowed the transformation of 1 µmol of substrate per minute. This procedure allowed the preparation of ChE solutions at 0.3 U/mL, which were stored at 4 °C before use.

#### 2.2.2. Fabrication of Screen-Printed Electrodes

A DEK 248 semi-automatic screen-printing machine was used to fabricate the electrodes according to a previously described method [11]. The resulting three-electrode system consisted of a 4 mm diameter carbon working electrode containing a cobalt phthalocyanine mediator, surrounded by a carbon counter electrode (16 mm × 1.5 mm), with a linear Ag/AgCl electrode (5 mm × 1.5 mm) acting as the reference electrode.

#### 2.2.3. Enzyme Immobilization

Either AChE or BChE enzymes were immobilized on the screen-printed electrode (SPE) sensor surface by entrapment in a polyvinyl alcohol gel. This immobilization technique has several advantages, including enhanced stability, as the polymer matrix protects the enzyme from external factors such as pH, temperature, and solvents, leading to enhanced thermal and chemical stability compared to free enzymes, as well as reduced enzyme leaching. Its selective permeability allows the diffusion of substrates and products while blocking larger interfering species, thus enhancing the selectivity of the biosensor compared to other techniques [12]. The enzyme solution containing 0.3 U/mL in PBS buffer at pH 7 was first mixed with polyvinyl alcohol photosensitive polymer (Biosurfine-MRH) in a 70%:30% ratio (*v*/*v*). Then, 3 µL of the resulting mixture was spread on the surface of the working electrode using a micropipette. The quantity of immobilized enzymes present was calculated to be 0.9 mU/electrode. The photopolymerization process was induced by placing the modified SPEs under 2 white neon lights (Philips T5 short, 4000 K, 8 W, 380 lm) for 48 h. The resulting biosensors were stored at 4 °C until further use.

#### 2.2.4. Biosensor Measurements

**Figure 1:**
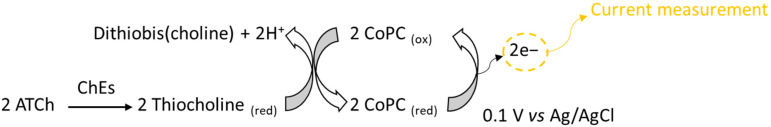
Principle of electrochemical detection of cholinesterase activity.

Chronoamperometric measurements were carried out in a 10 mL thermostatic glass cell. The biosensor was immersed in 10 mL of 0.1 M PBS buffer pH 7 [13] containing 0.1 M KCl, and a potential of 0.1 V versus Ag/AgCl was applied using a PG581 Uniscan potentiostat (Uniscan Instruments, Basildon, UK). The enzyme reaction was initiated upon the addition of 100 µL of 0.1 M acetylthiocholine chloride, and the oxidation current produced by CoPC mediator oxidation was measured at a steady state (Figure 1) [14]. The measurements were repeated four times to confirm the stability of the biosensor’s response. The same procedure was used to realize the measurements directly in milk.

For inhibition measurements, the biosensor was incubated for 10 min in 10 mL of PBS or milk containing quaternary ammonium biocides, and the residual response was measured as described above. The cell was washed with PBS between measurements. The initial responses were repeated four times in PBS and in whole, partially skimmed, and skimmed milk to confirm the stability of the biosensor’s response. The inhibition rate was calculated by comparing the biosensor responses before and after incubation with the biocide using the following equation: I_0_ − I_(biocide)_/I_0_, with I_0_ being the current response of the initial enzyme activity and I_(biocide)_ the activity of the enzyme in the presence of biocides. The biosensor calibration curves were established using known concentrations of BAC and DDAC. All the biosensors measurements were carried out in triplicate.

#### 2.2.5. Sample Pretreatment Using Liquid–Liquid Extraction

Stock solutions of BAC and DDAC at known concentrations were directly diluted in 1 mL of milk to achieve the desired final concentrations. For BAC, the stock solution was diluted to yield final concentrations ranging from 0.26 µM to 59 µM, while for DDAC, the stock solution was diluted to final concentrations ranging from 0.04 µM to 9 µM. This direct dilution approach ensured the accurate preparation of the spiked milk samples, maintaining the integrity of the matrix while allowing for precise control over the final biocide concentrations. The tubes were vortexed and allowed to rest for 10 min before the addition of 20 µL of formic acid at 85%. The solution was vortexed again and 9 mL of acetonitrile/ethylacetate (ACN/EtAc − 50:50) extraction solvent was added. The samples were vortexed once again to homogenize the mixture, and 2 g of MgSO_4_ was added to dry the aqueous phase. Subsequently, the tubes containing the samples were stirred for 10 min using a mechanical rotary shaker at 100 rpm to promote exchange between the sample and the extraction solvents. Finally, the samples were centrifuged at 4200 rpm for 10 min at 4 °C, and the supernatant was transferred into a flask. The solvent was evaporated at 50 °C using a rotary evaporator and the dry extract was diluted in 1 mL of water for electrochemical measurements. This pretreatment was previously described by members of the French Agency for Food, Environmental and Occupational Health & Safety (ANSES) [6].

## 3. Results and Discussion

### 3.1. Tests and Optimization of DM AChE- and BChE-Based Biosensors Operating in PBS

**Figure 2:**
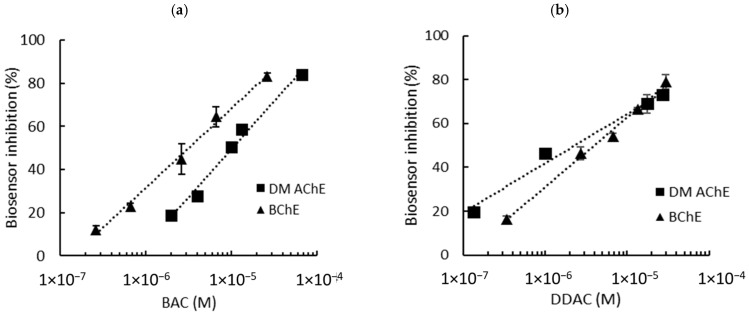
Inhibition effect of BAC (**a**) and DDAC (**b**) biocides on AChE- and BChE-based biosensors containing a 0.9 mU/electrode. The equations of the calibration curves are the following: (**a**) DM AChE: y = 19.27ln(x) + 271.23, R^2^ = 0.986; BChE: y = 15.95ln(x) + 251.96, R^2^ = 0.993; (**b**) DM AChE: y = 9.75ln(x) + 176.67, R^2^ = 0.983; BChE: y = 13.75ln(x) + 221.11, R^2^ = 0.993.

DM AChE- and BChE-based biosensor calibrations were conducted in PBS using standard concentrations of BAC and DDAC prepared in distilled water (Figure 2). The choice of these two enzymes was based on prior studies, which demonstrated that DM AChE and BChE exhibited the highest sensitivities for detecting DDAC and BAC, respectively [8]. Molecular docking and enzyme kinetics further revealed that while DDAC competitively inhibited both enzymes, BAC acted as a non-competitive inhibitor of BChE [15]. These distinct inhibition mechanisms, coupled with the structural differences between the enzymes, likely account for the observed variation in their amperometric responses.

Biosensor inhibition tests were carried out in presence various concentrations of biocides, ranging from 0.26 µM to 66 µM for BAC and 0.13 µM to 30 µM for DDAC. These concentrations were selected to induce enzyme inhibitions raging from approximately 15% to 80%. The graphs showing the inhibition of the biosensor’s response as a function of biocide concentration are presented in Figure 2a (BAC) and Figure 2b (DDAC). The limits of detection (LODs) of the biosensors were calculated using each curve equation as the biocide concentration, inducing a 10% decrease in the biosensor response. Interestingly, the two biosensors showed different sensitivities, with the DM AChE sensor being much more inhibited by DDAC.

As already explained, the sensors used in this study contain 0.9 mU of ChE per electrode. However, it is well known that the enzyme loading is a crucial parameter which strongly affects the sensitivity of the analysis [16]. Enzyme loadings of 0.3 mU per electrode were thus tested to evaluate the possibility of improving the sensors’ performance. The results are detailed in the Supplementary Information (Figures S1 and S2). As shown in Table 1, a decrease in the enzyme load did not lead to a significant decrease in the LOD. Moreover, the biosensors containing 0.3 mU of cholinesterase showed significant background noise compared to those containing 0.9 mU (Figure S2). Therefore, enzyme loadings of 0.9 mU per electrode were selected for further experiments. Using this loading, both DM AChE and BChE biosensors allowed for the detection of BAC and DDAC within the norms imposed by the EU, which sets a maximal concentration of 0.28 µM for milk products. It is interesting to note that DDAC strongly inhibited the DM AChE sensor, with a LOD of 0.04 µM, while BAC was more efficient at inhibiting the BChE sensor (LOD = 0.26 µM).

### 3.2. Detection of BAC and DDAC in Milk

#### 3.2.1. Direct Detection in Milk and the Effect of Fat Content

The promising LODs obtained in PBS buffer and their compatibility with European regulations suggest that it may be possible to detect BAC and DDAC in milk directly. However, the performance of enzymatic sensors is often affected by the need to dilute the sample in a selected buffered solution. To overcome this drawback, a simple alternative is to perform the measurements directly in the milk matrix. The first tests therefore consisted of measuring the response of the sensors in milk, without any dilution. Three types of milk were tested, including whole milk (WM), partially skimmed milk (PSM) and skimmed milk (SM), and the sensors’ responses were compared to those obtained in buffer (Table 2). Surprisingly, no significant change was observed in the responses of the DM AChE- and BChE-based biosensors when taking measurements in buffer or milk, suggesting the possibility of a direct detection of biocides in milk.

**Figure 3:**
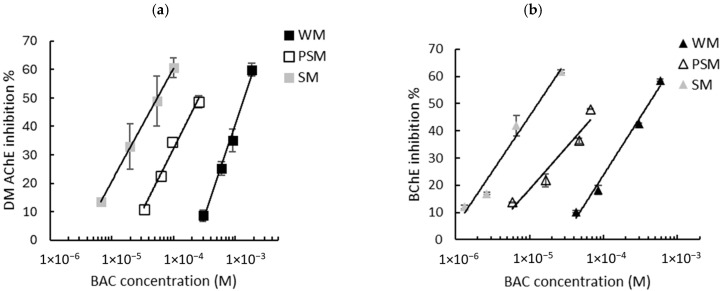
Inhibition effect of BAC biocide on (**a**) DM AChE- and (**b**) BChE-based biosensors. Measurements were carried out in whole milk (WM), partially skimmed milk (PSM), and skimmed milk (SM). The equations of the obtained curves are the following: (**a**) WM: y = 28.04ln(x) + 234.41, R^2^ = 0.989, PSM: y = 18.82ln(x) + 205.61, R^2^ = 0.985, SM: y = 17.3ln(x) + 219.82, R^2^ = 0.999; (**b**) WM: y = 18.57ln(x) + 194.88, R^2^ = 0.987, PSM: y = 13.43ln(x) + 173.27, R^2^ = 0.939, SM: y = 17.62ln(x) + 248.32, R^2^ = 0.971.

**Figure 4:**
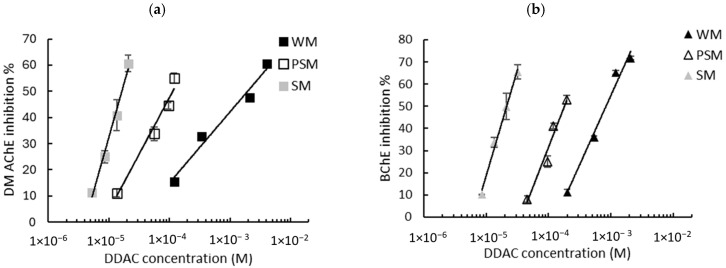
Inhibition effect of DDAC biocide on (**a**) DM AChE- and (**b**) BChE-based biosensors. Measurements were carried out in whole milk (WM), partially skimmed milk (PSM), and skimmed milk (SM). The equations of the obtained curves are the following: (**a**) WM: y = 11.79ln(x) + 123.54, R^2^ = 0.974, PSM: y = 18.81ln(x) + 220.76, R^2^ = 0.968, SM: y = 35.69ln(x) + 443.14, R^2^ = 0.989; (**b**) WM: y = 27.1ln(x) + 242.06, R^2^ = 0.981, PSM: y = 31.36ln(x) + 320.38, R^2^ = 0.960, SM: y = 41.38ln(x) + 495.08, R^2^ = 0.992.

Given that the intrinsic response of the biosensors is not influenced by the matrix, milk samples spiked with known concentrations of biocides were assayed and the sensors’ inhibition rates were determined. As described in Section 2.2.4, the biosensors’ responses were measured using either WM, PSM, or SM. The graphs showing the inhibition of the biosensors’ response as a function of the biocide concentration are presented in Figure 3 (BAC) and Figure 4 (DDAC). The results obtained show that the inhibition ratios obtained with BAC and DDAC were highly dependent on the type of milk analyzed. In each case, a decrease in the sensitivity of both biosensors was observed with an increasing fat content, suggesting a masking effect of fat matter on inhibition. This effect resulted in a shift in the calibration curves towards higher concentrations (Figure 3 and Figure 4). It was assumed that because of their amphiphilic nature, BAC and DDAC targets might be interacting with milk fat matter, leading to a reduction in their interaction with the enzyme and a decrease in biosensor inhibition.

The calibration curve equations obtained using either the DM AChE or BChE sensors in the three different milks allowed for the calculation of the LODs of the two biocides. A comparison of the LODs with those obtained in buffer is presented in Table 3. As previously observed in PBS buffer (Figure 2), a greater sensitivity was observed using DM AChE for DDAC and BChE for BAC. However, the sensors’ inhibition decreases sharply as the fat concentration increases, leading to a dramatic increase in the LODs. Even using skimmed milk, which contains less than 0.5% fat, the LOD is increased by a factor of between 4 and 36 when compared with the LOD obtained in PBS. This suggests that the decrease in sensor inhibition is not solely attributable to fat, but also to proteins (mainly caseins) that are present in milk in significant quantities, at between 3.3% (whole milk) and 3.6% (skimmed milk).

#### 3.2.2. Detection in PBS After Milk Treatment

Unfortunately, the results described above demonstrated that although cholinesterase activity is not intrinsically affected by the milk matrix, the inhibitory effect of the BAC and DDAC biocides was highly dependent on the type of milk analyzed. Moreover, when operating directly in milk, the developed biosensors were not able to detect BAC and DDAC at concentrations compatible with EU regulations (MRL = 0.28 µM).

**Figure 5:**
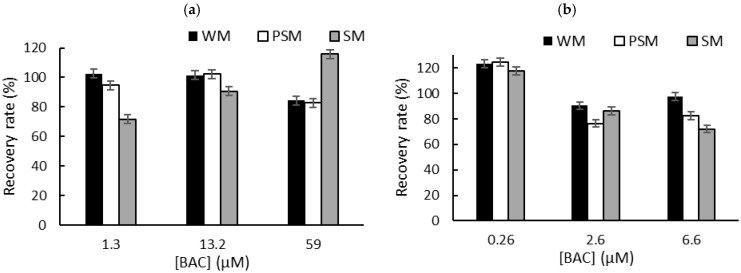
The recovery rates obtained for BAC after liquid–liquid extraction from whole milk (WM), partially skimmed milk (PSM), and skimmed milk (SM). The samples were spiked with BAC at concentrations corresponding to IC10 (LOD), IC50, and IC80. Measurements were carried out using (**a**) DM AChE- or (**b**) BChE-based biosensors.

**Figure 6:**
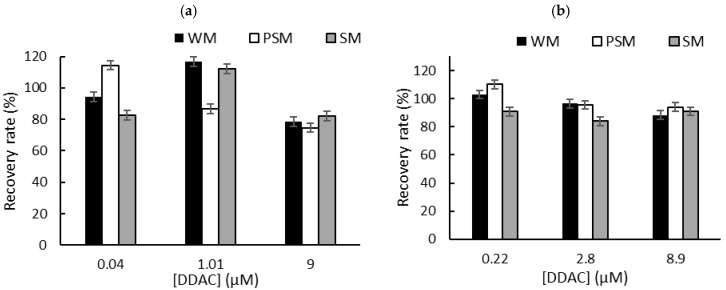
The recovery rates obtained for DDAC after liquid–liquid extraction from whole milk (WM), partially skimmed milk (PSM), and skimmed milk (SM). The samples were spiked with DDAC at concentrations corresponding to IC10 (LOD), IC50, and IC80. Measurements were carried out using (**a**) DM AChE- or (**b**) BChE-based biosensors.

To overcome the problem of the interactions between QA biocides and milk macromolecules (fats and proteins), we decided to carry out a liquid–liquid extraction prior to biosensor measurements in PBS [6]. Milk samples (1 mL) were spiked with known concentrations of biocide corresponding to the limit of detection (LOD), the concentration inducing 50% inhibition (IC50), and the concentration inducing 80% inhibition (IC80). These concentrations were selected according to the sensitivity of each sensor. After centrifugation, the supernatant was dried at 50 °C using a rotary evaporator, and the dry extracts containing BAC or DDAC biocides were diluted in 1 mL of distilled water. The resulting sample (1 mL) was added to a biosensor cell containing 9 mL of PBS, and electrochemical measurements were performed using DM AChE- and BChE-based sensors. A comparison with the initial signal measured in PBS buffer allowed for the calculation of the inhibition percentage, which was used to determine the recovered biocide concentration. The calculated recovery rates obtained for BAC (Figure 5) and DDAC (Figure 6) range from 71% to 120%, showing a good correlation between the expected and measured biocide concentrations. The graphs showing the concentrations measured by the biosensor versus the actual spiked concentration are presented in the Supplementary Information section (Figures S3 and S4). As expected, BAC was detected with higher sensitivity when using the BChE-based biosensor, validating the LOD of 0.26 µM (Figure 5b). The DM AChE biosensor showed a lower sensitivity but was able to detect BAC with good reliability over a wider range of concentrations, up to 59 µM (Figure 5a). In contrast, the DM AChE biosensor showed a higher sensitivity to DDAC, with a LOD as low as 0.04 µM (Figure 6a). Both biosensors allowed for the reliable determination of DDAC at concentrations up to 9 µM (Figure 6), keeping in mind that the maximum concentration tolerated in food industry dairy products is 0.28 µM. However, in the case of BAC, only the BChE-based biosensor was sensitive enough to comply with EU regulations.

## 4. Conclusions

In this study, we successfully developed and validated electrochemical enzymatic sensors for the detection of BAC and DDAC biocides in whole, partially skimmed, and skimmed cow milk. Despite the challenging matrix, the biosensors were able to operate directly in milk without apparent interference; however, significant matrix effects were observed when conducting inhibition measurements. These effects resulted in incorrect determinations of BAC and DDAC due to the presence of milk fats and proteins. To overcome this problem, a liquid–liquid extraction was performed to efficiently extract biocides from the milk matrix. This pretreatment step allowed us to perform electrochemical measurements with improved sensitivity and accuracy. However, only the BChE-based biosensor was capable of detecting both biocides at concentrations compatible with European regulations, with LODs of 0.23 µM and 0.26 µM for DDAC and BAC, respectively.

Building upon these findings, future work will focus on optimizing the extraction process of complex matrices such as milk to make the method more efficient and adaptable for on-site or real-time monitoring applications while preserving the integrity of the analytes. Another perspective could be to combine the two biosensors into a unique detection system, allowing biocides to be detected not only sensitively but also specifically, using data analysis based on machine learning.

## Figures and Tables

**Table 1:** Limits of detection (µM) obtained for BAC and DDAC using biosensors containing either 0.3 or 0.9 mU of cholinesterase (LODs were calculated at a 10% inhibition rate, using the equations described in Figure 2, Figure S1 and Figure S2).

	DM AChE		BChE	
	0.3 mU	0.9 mU	0.3 mU	0.9 mU
BAC	0.98	1.3	0.21	0.26
DDAC	0.04	0.04	0.1	0.22

**Table 2:** Relative responses of the two biosensors operating in non-diluted whole milk (WM), partially skimmed milk (PSM) and skimmed milk (SM) in comparison with the standard responses measured in PBS. The enzyme response was obtained in presence of ATCh at 1 mM.

		PBS	WM	PSM	SM
Relative response (%)	DM AChE biosensor	100	99.4	106.2	89.1
	BChE biosensor	100	105.6	101	90.5

**Table 3:** Limits of detection (LODs) (µM) of BAC and DDAC biocides calculated for DM AChE- and BChE-based biosensors operated in whole milk, partially skimmed milk, and skimmed milk. The LODs obtained in PBS were added for comparison.

	DM AChE		BChE	
	BAC	DDAC	BAC	DDAC
Whole milk	335.1	65.8	47.5	119.2
Partially skimmed milk	30.7	13.6	5.3	50.4
Skimmed milk	5.4	5.0	1.3	8.1
PBS (0.1 M pH7)	1.3	0.04	0.26	0.22

## Data Availability

Data are contained within the article.

## Supplementary Materials

The following supporting information can be downloaded at https://www.mdpi.com/article/10.3390/bios15010026/s1: Figure S1: The effect of biocide concentration on biosensors containing 0.3 mU of ChE: (a) inhibition by BAC, (b) inhibition by DDAC. The equations of the obtained curves are the following: (a) DM AChE: y = 17.622ln(x) + 253.81, (R^2^ = 0.984); BChE: y = 15.287ln(x) + 244.83, (R^2^ = 0.861) and (b) DM AChE: y =9.1511ln(x) + 166.92, (R^2^ = 0.947); BChE: y = 12.39ln(x) + 209.81, (R^2^ = 0.962). Figure S2: The Effect of biocide concentration on biosensors containing 0.9 mU of ChE: (a) inhibition by BAC, (b) inhibition by DDAC. The equations of the obtained curves are the following: (a) DM AChE: y = 17.925ln(x) + 255.68, (R^2^ = 0.984); BChE: y = 15.974ln(x) + 248.49, (R^2^ = 0.986) and (b) DM AChE: y = 10.904(x) + 191.85, (R^2^ = 0.977); BChE: y = 13.649ln(x) + 218.02, (R^2^ = 0.996). Figure S3: Representation of BAC recovery concentrations vs. BAC real concentrations in whole milk (WM), partially skimmed milk (PSM), and skimmed milk (SM) samples using (a) DM AChE-based biosensors and (b) BChE-based biosensors. Figure S4: Representation of DDAC recovery concentrations vs. DDAC real concentrations in whole milk (WM), partially skimmed milk (PSM), and skimmed milk (SM) samples using (a) DM AChE-based biosensors and (b) BChE-based biosensors.

## Abbreviations

DM AChE	Acetylcholinesterase from *Drosophila melanogaster*
ANSES	Agency for Food, Environmental and Occupational Health & Safety
BAC	Benzalkonium chloride
BChE	Butyrylcholinesterase from horse serum
PVA	Biosurfine-MRH photopolymer
CoPC	Cobalt phthalocyanine
ChE	Cholinesterase
DDAC	Didecyldimethylammonium chloride
DTNB	5,5′-dithiobis-2-nitrobenzoic acid
EU	European Union
EC	European Commission
LOD	Limits of detection
LOQ	Limits of quantification
MRL	Maximum residue limit
PBS	Phosphate-buffered saline
PSM	Partially skimmed milk
PVC	Poly (vinyl) chloride
QAs	Quaternary ammonium salts
SM	Skimmed milk
SPE	Screen printed electrode
TNB	5-thio-2-nitrobenzoic acid
WM	Whole milk
