# Supplementary material for: Enhancing Biocide Safety of Milk Using Biosensors Based on Cholinesterase Inhibition
Source: Biosensors (Basel). 2025 Jan 6;15(1):26. doi: 10.3390/bios15010026 (PMC11763983; doi:10.3390/bios15010026)
Supplement: Supplementary file 1 [file biosensors-15-00026-s001.zip › biosensors-3324629-supplementary.pdf]

Supplementary data

Enhancing Biocide Safety of Milk Using Biosensors Based on Cholinesterase Inhibition

Lynn Mouawad [1,2], Georges Istamboulie [1,2], Gaëlle Catanante [1,2,*] and Thierry Noguer [1,2,*]

[1] Biosensors Analysis Environment Group (BAE-LBBM), Université de Perpignan, Via Domitia, 52 Avenue Paul Alduy, Cedex, F-66860 Perpignan, France
[2] Laboratoire de Biodiversité et Biotechnologie Microbienne (LBBM), Sorbonne Université, Observatoire Océanologique, F-66650 Banyuls-sur-Mer, France

(a) (b)

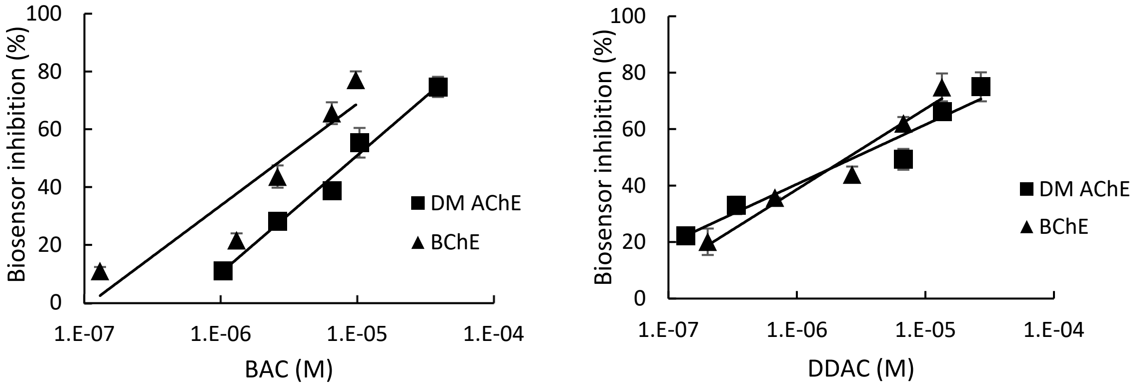

Figure S1: Effect of biocide concentration on biosensors containing 0.3 mU of ChE (a) inhibition by BAC, (b) inhibition by DDAC. Equations of the obtained curves are the following: (a) DM AChE: y= 17.622ln(x) + 253.81, (R^2=0.984); BChE: y=15.287ln(x) + 244.83, (R^2=0.861), (b) DM AChE: y=9.1511ln(x) + 166.92, (R^2=0.947); BChE: y=12.39ln(x) + 209.81, (R^2=0.962).

(a) (b)

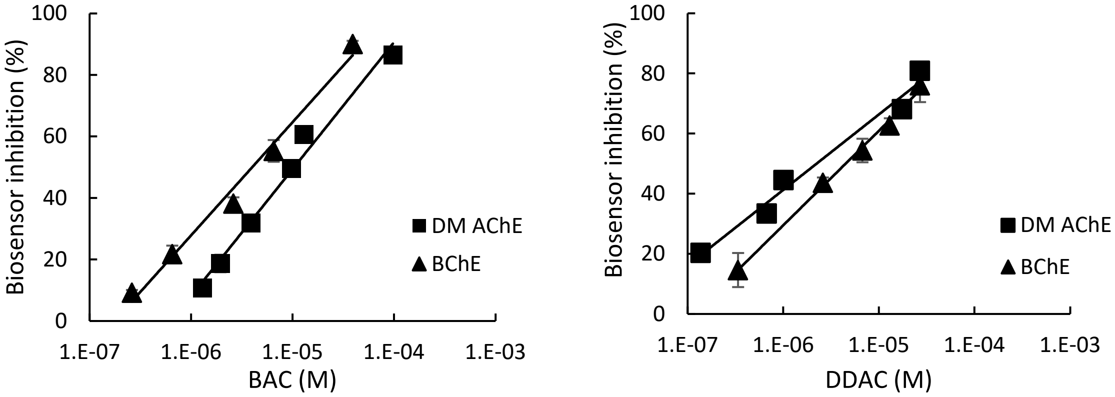

Figure S2: Effect of biocide concentration on biosensors containing 0.9 mU of ChE (a) inhibition by BAC, (b) inhibition by DDAC. Equations of the obtained curves are the following: (a) DM AChE: y= 17.925ln(x) + 255.68, (R^2=0.984); BChE: y=15.974ln(x) + 248.49, (R^2=0.986). (b) DM AChE: y=10.904(x) + 191.85, (R^2=0.977); BChE: y= 13.649ln(x) + 218.02, (R^2=0.996).

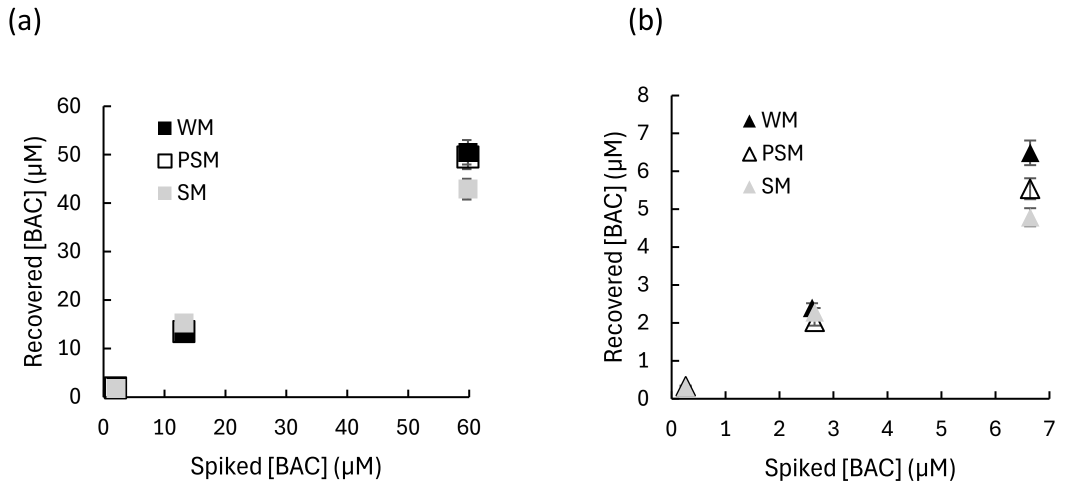

Figure S3: Representation of BAC recovery concentrations vs BAC real concentrations in whole milk (WM), partially skimmed milk (PSM), and skimmed milk (SM) samples using (a) DM AChE based biosensor and (b) BChE based biosensor.

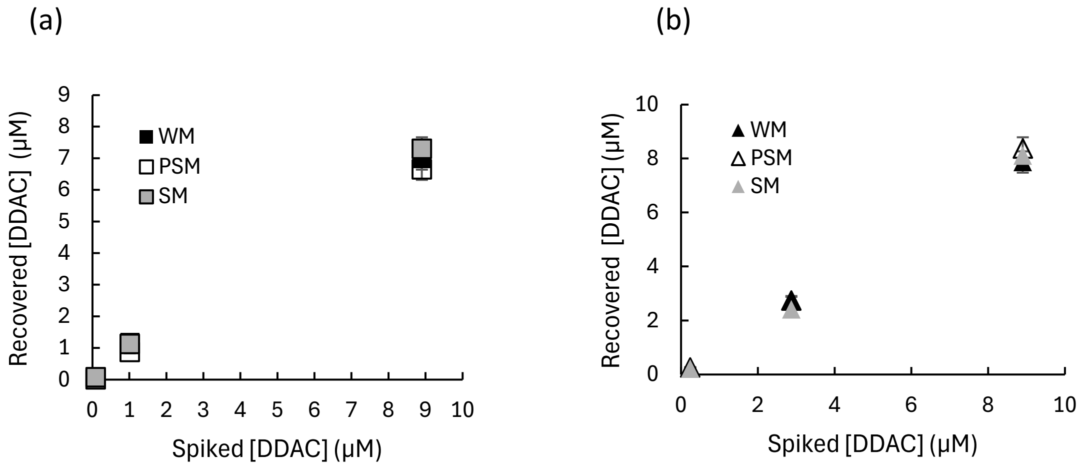

Figure S4: Representation of DDAC recovery concentrations vs DDAC real concentrations in whole milk (WM), partially skimmed milk (PSM), and skimmed milk (SM) samples using (a) DM AChE based biosensor and (b) BChE based biosensor.
